# Study on Large Deformation Behavior of Polyacrylamide Hydrogel Using Dissipative Particle Dynamics

**DOI:** 10.3389/fchem.2020.00115

**Published:** 2020-02-25

**Authors:** Jincheng Lei, Shuai Xu, Ziqian Li, Zishun Liu

**Affiliations:** International Center for Applied Mechanics, State Key Laboratory for Strength and Vibration of Mechanical Structures, Xi'an Jiaotong University, Xi'an, China

**Keywords:** polyacrylamide hydrogel, dissipative particle dynamics, large deformation behavior, effective network, fracture criterion

## Abstract

Meso-scale models for hydrogels are crucial to bridge the conformation change of polymer chains in micro-scale to the bulk deformation of hydrogel in macro-scale. In this study, we construct coarse-grain bead-spring models for polyacrylamide (PAAm) hydrogel and investigate the large deformation and fracture behavior by using Dissipative Particle Dynamics (DPD) to simulate the crosslinking process. The crosslinking simulations show that sufficiently large diffusion length of polymer beads is necessary for the formation of effective polymer. The constructed models show the reproducible realistic structure of PAAm hydrogel network, predict the reasonable crosslinking limit of water content and prove to be sufficiently large for statistical averaging. Incompressible uniaxial tension tests are performed in three different loading rates. From the nominal stress-stretch curves, it demonstrated that both the hyperelasticity and the viscoelasticity in our PAAm hydrogel models are reflected. The scattered large deformation behaviors of three PAAm hydrogel models with the same water content indicate that the mesoscale conformation of polymer network dominates the mechanical behavior in large stretch. This is because the effective chains with different initial length ratio stretch to straight at different time. We further propose a stretch criterion to measure the fracture stretch of PAAm hydrogel using the fracture stretch of C-C bonds. Using the stretch criterion, specific upper and lower limits of the fracture stretch are given for each PAAm hydrogel model. These ranges of fracture stretch agree quite well with experimental results. The study shows that our coarse-grain PAAm hydrogel models can be applied to numerous single network hydrogel systems.

## Introduction

A hydrogel is a network of polymer chains swollen in water. Synthetic hydrogels have developed rapidly since the landmark research by Wichterle and Lím (1960). Due to the large water content, hydrogels can be bio-compatible, highly compliant and exhibit low friction, making them widely used for personal care and medical applications, such as superabsorbent diapers (Masuda, 1994), contact lenses (Caló and Khutoryanskiy, 2015), drug delivery (Li and Mooney, 2016; Liu et al., 2017), tissue engineering (Lee and Mooney, 2001; Haque et al., 2012; Lienemann et al., 2012; Xu et al., 2018), and wound dressing (Li et al., 2017). However, the large water content also makes hydrogels have very low resistance to deformation and fracture (Zheng et al., 2018; Xu and Liu, 2019) and hard to be used as load-bearing structures. Since Gong's (Gong et al., 2003) work, diverse efforts have been made to design tough hydrogels by building dissipations into the networks (Zhao, 2014). Examples include double-network hydrogels (Gong et al., 2003; Henderson et al., 2010), poly(vinyl alcohol) hydrogels with crystalline domains (Peppas and Merrill, 1976; Stauffer and Peppast, 1992), hydrogels with hybrid physical and chemical crosslinkers (Kong et al., 2003; Shull, 2012; Sun et al., 2012), and hydrogels with transformable domains (Brown et al., 2009). It is well-known that the hyper-elastic nature of hydrogel originates from its crosslinking polymer network (Liu Z. et al., 2015). Yet the crosslinking polymer network in hydrogels still stays in the realm of hypothesis since the dynamic experimental observations and determinations are inconvenient at such a micro level. In order to reveal the true nature of the crosslinking polymer network during the deformation and fracture of hydrogels, we have to bring ourselves down to the mesoscale or even molecular scale, where molecular dynamics (MD) simulations and Monte-Carlo simulations could be an effective approach.

Researchers have proposed all-atom models (Tönsing and Oldiges, 2001; Oldiges and Tönsing, 2002; Wu et al., 2009; Mathesan et al., 2016; Xu et al., 2016; Hou et al., 2019) to investigate the structural and physical properties of hydrogels, such as the hydrogen-bond configuration and thermal conductivity. These models usually contain only several short polymer chains whose lengths are in the same scale of the persistent length of polymer chains. This makes them hard to depict the compliant polymer network in hydrogels. Besides, most researches focus on the equilibrium properties of hydrogels without bearing loads. An et al. (2019) investigated the mechanical properties of hydrogels via MD simulations, but the strain is small. Jang et al. (2007) obtained the stress-strain curve under large strain, but the stress level is several orders of magnitude larger (as well as An et al.'s work) than real situations. Although many general force fields, including consistent valence force field (CVFF) (Dauber-Osguthorpe et al., 1988), optimized potentials for liquid simulations (OPLS) force field (Kaminski et al., 1994), GROMACS force field (Berendsen et al., 1995), DREIDING force field (Mayo et al., 1990), etc., have been proposed and can be applied to the polymer-solvent system, the sophisticated atomic models with too many structural details hinders the extension of length scale and time scale. Since the large deformation behavior of hydrogels originates from the conformation change of the polymer network in mesoscale, a coarse-grain model of hydrogels balancing the structural features and computational time is in great need.

Dissipative particle dynamics (DPD) (Hoogerbrugge and Koelman, 1992; Koelman and Hoogerbrugge, 1993) is a mesoscale particle method that bridges the gap between macroscopic and microscopic simulations. Combining with coarse-grain methods, it is very suitable for simulating gaseous or fluid systems. DPD has been successfully applied to diverse areas of interests, especially to simulate the equilibrium and dynamical properties of polymers in solution as well as polymer gels (Spenley, 2000; Maiti and McGrother, 2004; Symeonidis et al., 2005; Zhao, 2014). When applying DPD, or other coarse grained MD simulations to study polymers or polymer gels, a physical crosslinking process is crucial to construct real polymer network. However, many previous works (Wu et al., 2009; Nawaz and Carbone, 2014; Mathesan et al., 2016; Jin et al., 2018; Hou et al., 2019; Xing et al., 2019) construct polymer models based on hypothetical cross-linked network structures which inevitably loses some structural components, such as branch chains, polymer loops, unreacted monomers, short segments et al. For instance, Xing et al. (2019) constructed 3D cross-linked networks for DNA hydrogels, while the chain length between cross-linking points was set to be constant. Jin et al. (2018) built randomly cross-linked polymer networks with the real cross-linking densities, while all the polymer chains are forced to crosslink.

In this study, the polyacrylamide hydrogel is taken to be the representative material. We build the bead-spring models of PAAm hydrogel by using DPD to simulate the crosslinking process from the mixture of monomers, cross-linkers and water. Details on the model construction are discussed in section Materials and Methods. The large deformation and fracture behavior of our PAAm hydrogel models are discussed in section Results.

## Materials and Methods

In this section, a series of bead-spring models for PAAm hydrogel are constructed and tested using DPD simulations. In addition, a full-atom model for PAAm chain is also constructed and tested using classical MD simulations to provide the fracture criterion of the polymer chains in DPD simulations. All these simulations are performed in large-scale atomic/molecular massive parallel simulator (LAMMPS) (Plimpton, 1995).

### Governing Equation of DPD Simulations

DPD method shares the common features of coarse-grained MD as the larger length scale and time scale by predigesting the exquisite atomic interactive force in classical MD. A softer interactive force between particles is adopted in DPD simulations and divided into three parts as (Groot and Warren, 1997)

$$\begin{array}{l} {\;\vec{F}}_{i}=\underset{j\neq i}{\sum }{\vec{F}}_{ij}^{C}+{\vec{F}}_{ij}^{D}+{\vec{F}}_{ij}^{R}\\ \;F_{ij}^{C}=a_{ij}\omega \left(r_{ij}\right),\;F_{ij}^{D}=-\gamma \omega^{2}\left(r_{ij}\right)\left({\hat{r}}_{ij}\cdot {\vec{v}}_{ij}\right),\\ \;F_{ij}^{R}=\sigma \omega \left(r_{ij}\right)\alpha {\left(\Delta t\right)}^{-\frac{1}{2}}\\ \omega \left(r_{ij}\right)=\{\begin{array}{c} 1-\frac{r_{ij}}{R_{c}},\;r_{ij}\leq R_{c}\\ 0,\;r_{ij}>R_{c} \end{array} \end{array}$$

where *r*_*ij*_ is distance between two beads. The $F_{ij}^{C}$ is the conservative force. It acts as the repulsive force between two particles within the cutoff *R*_*c*_, which is linearly related to the particle distance with the repulsive coefficient *a*_*ij*_. The $F_{ij}^{D}$ is the dissipative force which always points to the opposite direction of the relative velocity ${\vec{v}}_{ij}$ along the center line of two particles. It represents the effect of viscosity, depending on a coefficient γ and the distance between particles. The $F_{ij}^{R}$ is the random force. It also depends on a coefficient $\sigma =\sqrt{2kT\gamma }$ and the distance between two particles as well as a random variable α with the standard normal distribution. Since the dissipative force and random force act as the energy sink and source, the balance between two forces as a thermostat can be ensured by σ = 3, following the Groot's (Groot and Warren, 1997; Groot and Rabone, 2001) work.

Besides, it is convenient to normalize the mass of particles, the energy and the length of interactive cutoff as non-dimensional *m* = *kT* = *R*_*c*_ = 1, where *k* is the Boltzmann constant and *T* the temperature. Thus, the Newton's second law governing the particles motion is expressed by $\frac{d^{2}r_{i}^{⇀}}{dt^{2}}={\vec{F}}_{i}$. Meanwhile, the unit temperature *T* = 1 in normalized DPD refers to absolute temperature 300 K. The time unit in DPD simulations is also normalized as $\tau =\sqrt{mR_{c}^{2}/kT}=\;1$.

An additional bonding force acting as the bonds between polymer beads is described as

$$\begin{array}{l} {\vec{F}}_{i}^{B}=\underset{j}{\sum }C\left(r_{ij}-r_{0}\right){\hat{r}}_{ij}\; \end{array}$$

where *C* is the bond coefficient. A soft bond coefficient 4.0 (Rao et al., 2014; Wei et al., 2017) is used in the crosslinking simulations, while *C* = 116, 000 is adopted in tension simulations to match the real ratio of C-C bond strength to the heat fluctuation. *r*_0_ is the equilibrium bond length between two polymer beads. We take the mean distance between two beads *r*_0_ = *R*_*c*_ = 1.

### Coarse-Graining

**Figure 1:**
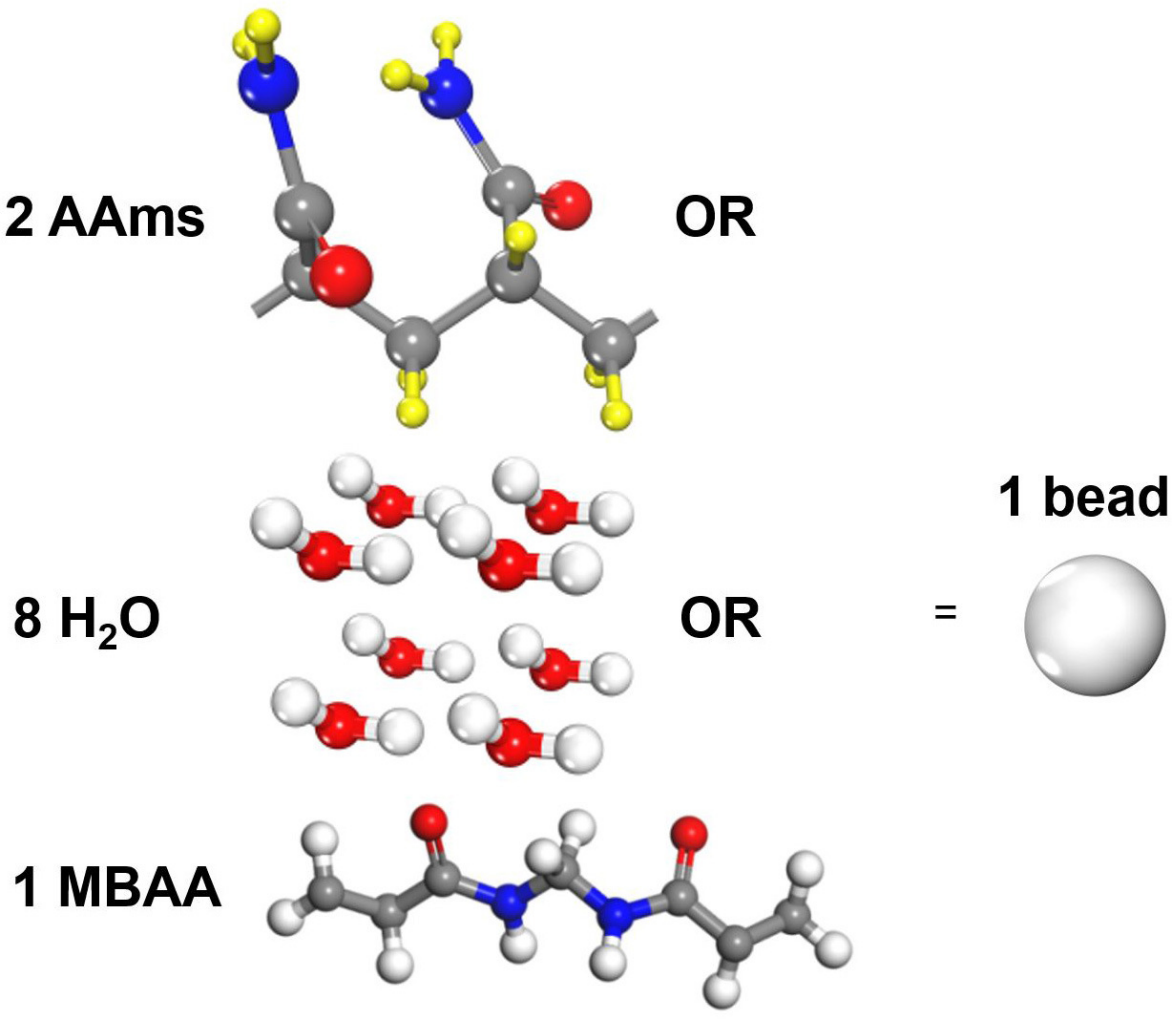
Coarse-grain scale of the polyacrylamide hydrogel model (One AAm bead refers to two connected AAm monomers. One water bead refers to eight water molecules. One MBAA bead refers to one MBAA molecule).

Three types of molecules are coarse-grained in our study, i.e., acrylamide(AAm), methylenebisacrylamide (MBAA) and water as shown in Figure 1. Since the mass of all particles in DPD simulations are considered to be the same, one AAm bead refers to two AAm monomers, one MBAA bead refers to one MBAA molecules, and one water bead refers to eight water molecules, to make sure their relative molecular weights are close. The validity of a coarse-grained model is determined by the interactive parameters in force field. According to Groot and Warren (1997) and Liu M. B. et al. (2015), the repulsive coefficient *a* for the same type of beads can be approximated by

$$\begin{array}{l} a_{ii}=\frac{75kT}{\rho } \end{array}$$

where ρ is the number of beads in the volume of $R_{c}^{3}$. Previous researchers chose ρ = 3 to get a better match on the compressibility of the model fluid (Liu M. B. et al., 2015). However, this high number density of beads leads to pure repulsion, because the mean distance between beads is much smaller than the repulsion cutoff *R*_*c*_ in conservative force *F*^*C*^. It makes the model solution under very high hydrostatic pressure when the number density is higher than $\sqrt{2}$ for the closest packing beads. Here we choose $\rho =\sqrt{2}$ to simulate the crosslinking process only affected by the viscosity and heat fluctuations, where the repulsion force between closest packing beads just vanishes. For different types of beads, Groot (Groot and Warren, 1997; Groot and Rabone, 2001) suggest the repulsive coefficient *a*_*ij*_ is linearly related to the Flory-Huggins parameters χ_*ij*_ with *a*_*ij*_ = *a*_*ii*_ + 3.27*kTχ*_*ij*_, where the χ_*ij*_ characterizes the mixture energy needed to form an equilibrium interaction between two clusters. We assume the monomer beads and crosslinker beads are the same type of beads denoted as polymer beads, and we take the repulsive coefficient χ_*ij*_ = 0.57 between polymer beads with water beads (Wei et al., 2017).

**Figure 2:**
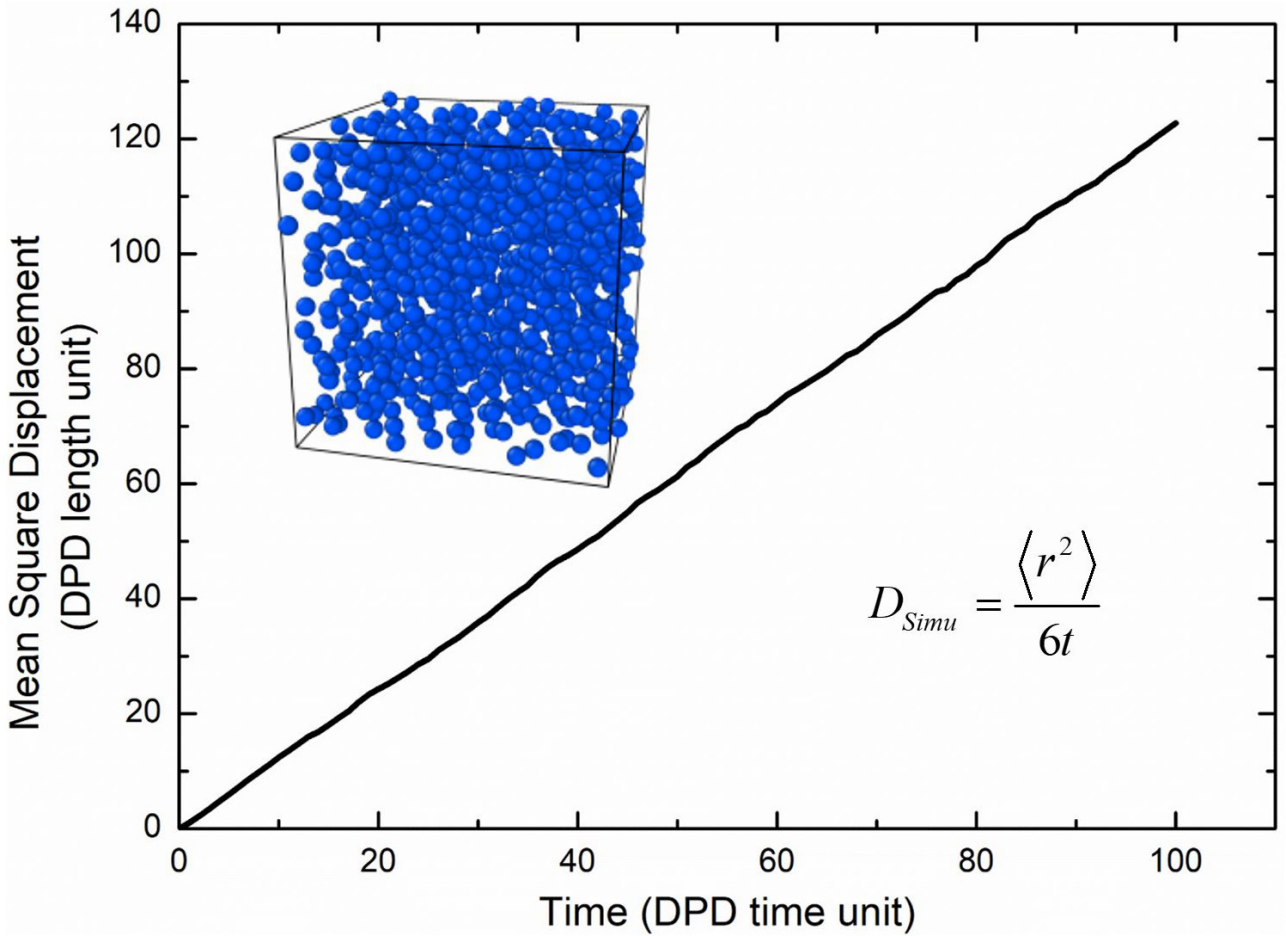
Self-diffusion of water beads.

The real time scale of a DPD time unit can be estimated by comparing the diffusivity of water beads with the real diffusivity of water as follows (Groot and Rabone, 2001)

$$\begin{array}{l} \tau =\frac{N_{m}D_{simu}R_{cr}^{2}}{D_{water}}, \end{array}$$

where the *N*_*m*_ is the coarse-grained scale representing the water molecule number that a water bead is, *D*_*water*_ the real diffusivity of water 2.43 ×10^−9^ *m*^2^/*s*, *R*_*cr*_ the real length of the DPD cutoff which can be obtained by the real volume of one water beads as 6.98 Å. The simulated diffusivity of water *D*_*simu*_ can be obtained from three independent simulations and calculated by $D_{simu}=\frac{\langle r^{2}\rangle }{6t}=0.212\pm 0.002$ as shown in Figure 2, where 〈*r*^2^〉 is the mean square displacement of all water beads. Therefore, the real time for DPD unit time is about 340.0 ps.

### Modeling and Testing

As mentioned in introduction, most current models of polymer network are constructed based on the theoretical hypothesis, such as full crosslinking, crosslinking with certain orientation or certain degree of polymerization etc. However, the real polymer networks in PAAm hydrogels are mostly imperfect. In order to reveal the real conformation of the polymer network, we simulate the crosslinking process of the precursor solution. The crosslinking process of PAAm hydrogel in experiments often lasts hours because it is a growth of polymer chains guided by initiator (Sun and Marshall, 1981), such as tetramethylethylenediamine (TEMED) (Bai et al., 2017; Tang et al., 2017; Zhang et al., 2018; Lei et al., 2019). Although the time scale of DPD simulation is increased by coarse-grain method, it still cannot affords the full simulation of real crosslinking process to capture both the growth of polymer chains and the diffusion of monomers and crosslinkers in precursor solution. However, when the amount of initiator is close to the magnitude of precursor, the growth of polymer chains starts from everywhere in precursor solution, so that it can be simply regarded as the simultaneously bonding process as long as reactant molecules are close enough. Moreover, if we consider unreacted molecules to be identical in the precursor solution, it would not make much difference on the crosslinking polymer network when the diffusion length of unreacted molecules is much larger than their mean distance within our simulation time.

Based on the assumptions above, we conduct the following crosslinking simulations. Random mixture models with water content 80% are built and crosslinked under different temperatures. Temperature in DPD simulations determines the diffusion length of beads. Low temperature with insufficient diffusion length leads to localized polymerization in which it's hard to form an effective polymer network throughout hydrogel. In order to find the proper temperature for crosslinking in DPD simulations, we choose six temperatures, i.e., *T* = 1, 3, 5, 7, 9, 11, to investigate the effect of diffusion length on the crosslinking process. Meanwhile, the effect of different water contents on the crosslinking process is investigated by simulating the crosslinking process of random mixture models with different water content 99, 98, 97, 96, and 95%. These high water contents are chosen to find the lowest crosslinking threshold of precursor content.

**Figure 3:**
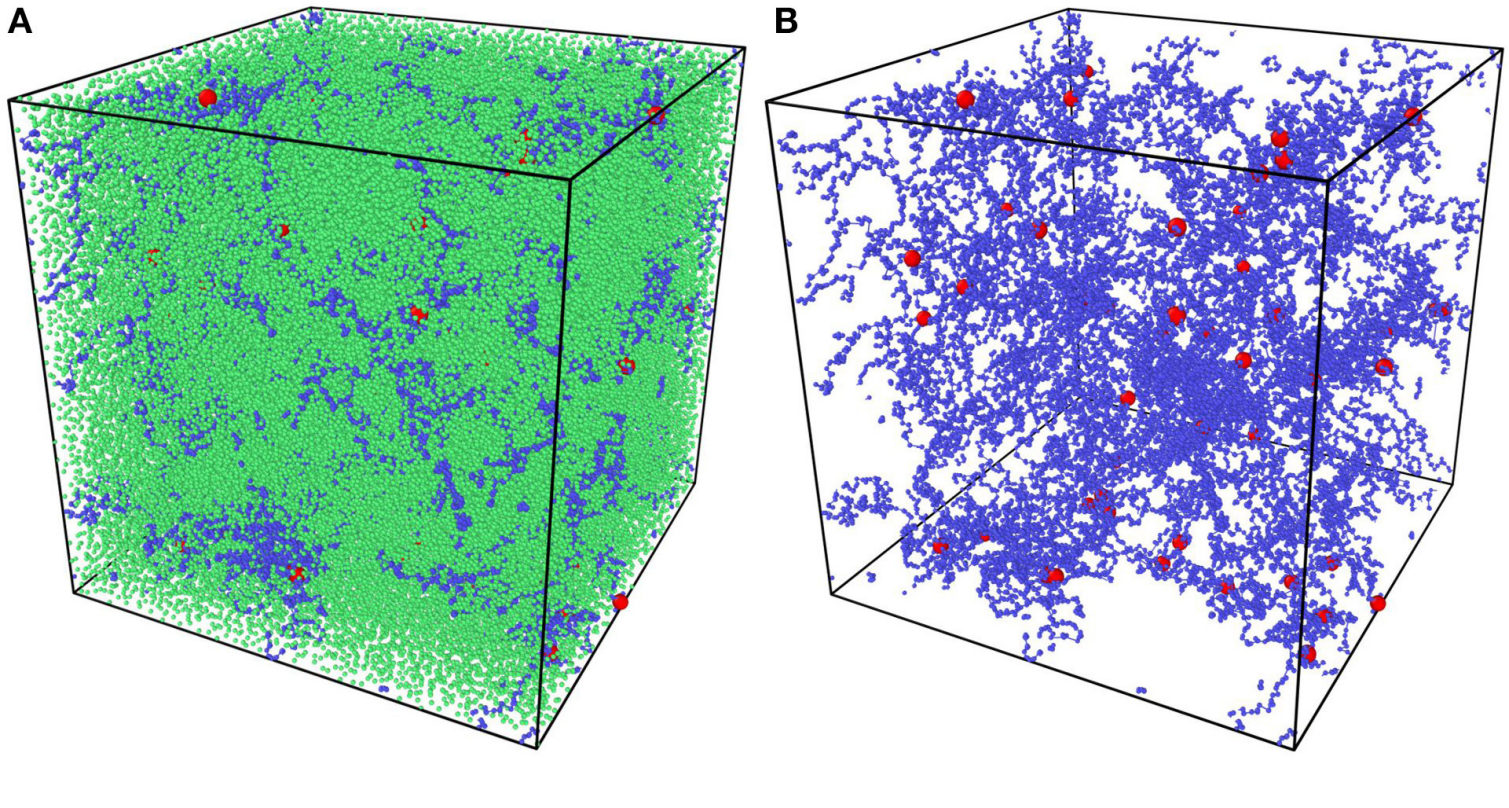
**(A)** The coarse-grain PAAm hydrogel model. **(B)** The coarse-grain PAAm hydrogel model without water beads (Blue beads are AAm beads. Red beads are MBAA beads. Green beads are water beads).

All random mixture models are composed of 125,000 beads with the certain precursor mass ratio AAm:MBAA = 1:0.002. For example, models with water content 80% have 100,000 water beads, 24,954 AAm beads and 46 MBAA beads. The simulation box size is ${\left(44.54R_{c}\right)}^{3}$. NVT ensemble is adopted as the thermostat. Time step is set as 0.01 DPD time. The mixture models are relaxed for 10,000 time steps first. Then the crosslinking process is performed by creating bonds between polymer beads every 10 time steps within 500,000 time steps, when the distance between two polymer beads is smaller than one DPD cutoff *R*_*c*_. The crosslinked models are then cooled down to *T* = 1 within 100,000 time steps and relaxed for another 100,000 time steps. For each water content and temperature, three independent models are generated for statistical averaging. Figure 3A is one of the models and Figure 3B show its polymer network without water beads, where blue beads are AAm beads, red beads are MBAA beads and green beads are water beads. All the following figures about the model structure are shown without water beads for better view.

Polymer chains in all models are classified into different chain types for structural analysis. Incompressible uniaxial tension tests are simulated in NVT ensemble with *T* = 1 in three loading rates 0.005, 0.001, and 0.0002 per DPD time unit. It should be noted that the loading rates here are true strain rate for the convenience of the implementation of incompressible uniaxial tension in LAMMPS. Thus, the true strain rates in other two directions are set to be half of the loading rate. Time step for tension tests is set as 0.001 DPD time.

### Fracture Criterion of Polyacrylamide Chain

**Figure 4:**
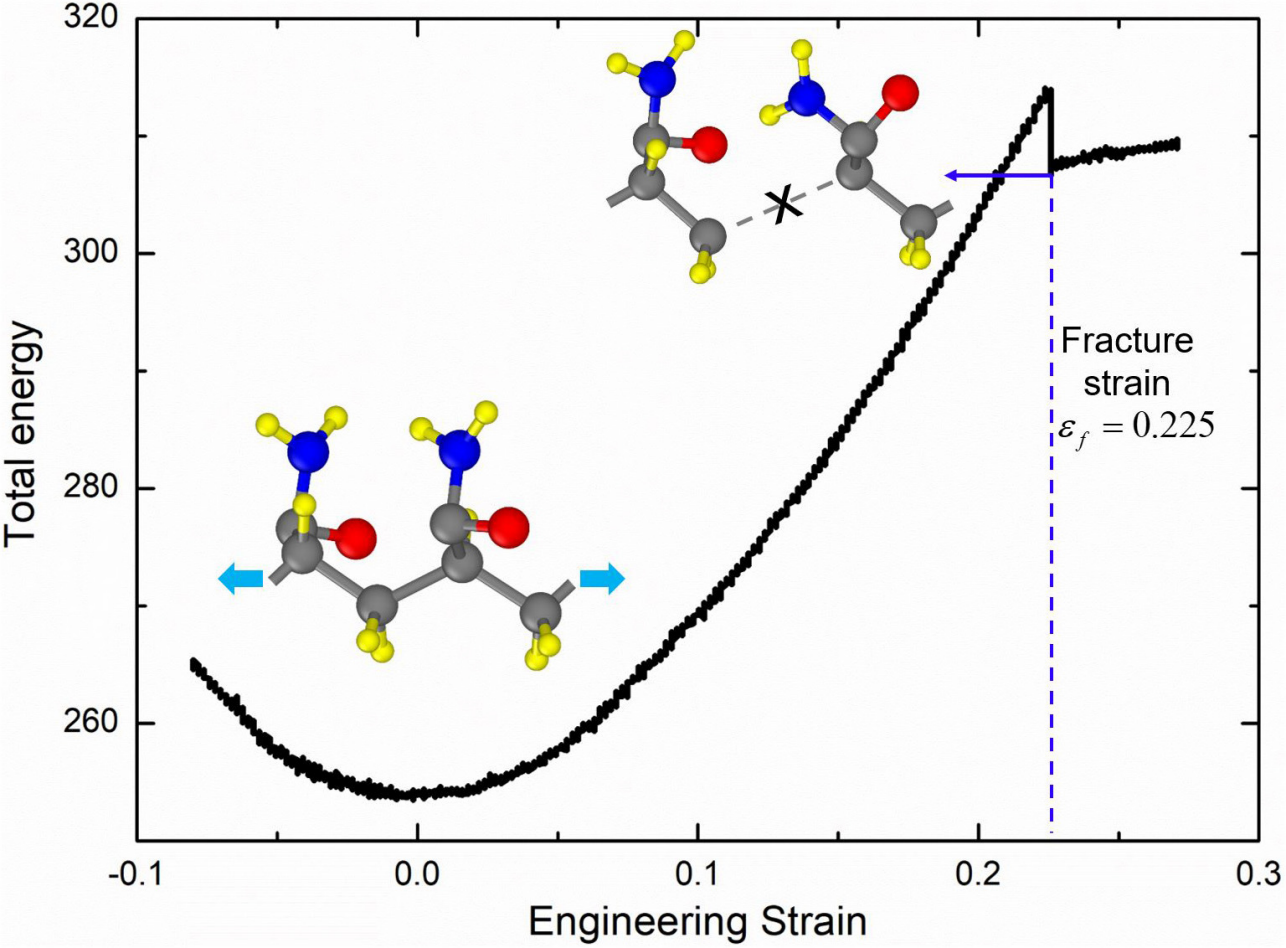
The energy-strain curve of the PAAm chains (The PAAm chain model is composed of two AAm monomers. The fracture strain is 0.225).

The large deformation of PAAm hydrogel is dominated by the polymer network conformation change in mesoscale, while the fracture of polymer chains is determined by the bond break in atomic scale. In order to obtain the fracture criterion of polymer chains in PAAm hydrogel, we build a full-atom model for PAAm chains with two connected AAm monomers as shown in the inset in Figure 4. This model corresponds to one AAm bead in our DPD simulations. Consistent valence force field (CVFF) (Dauber-Osguthorpe et al., 1988) is adopted to characterize the electrostatic forces, van der Waals interactions, bonds, bond angles, dihedrals and impropers between atoms. Detailed force field parameters can be found in Supplementary Material. Tension is imposed along chain direction in NVT ensemble with the loading rate 0.01/ps and temperature *T* = 300*K*. The time step is set as 0.1 fs. The energy-strain curve in Figure 4 shows the fracture of AAm chains occurs when the engineering strain is 0.225. This strain is chosen to be the fracture criterion we use to analyze the fracture properties of PAAm hydrogel models in DPD simulations.

## Results

### Model Validation

**Figure 5:**
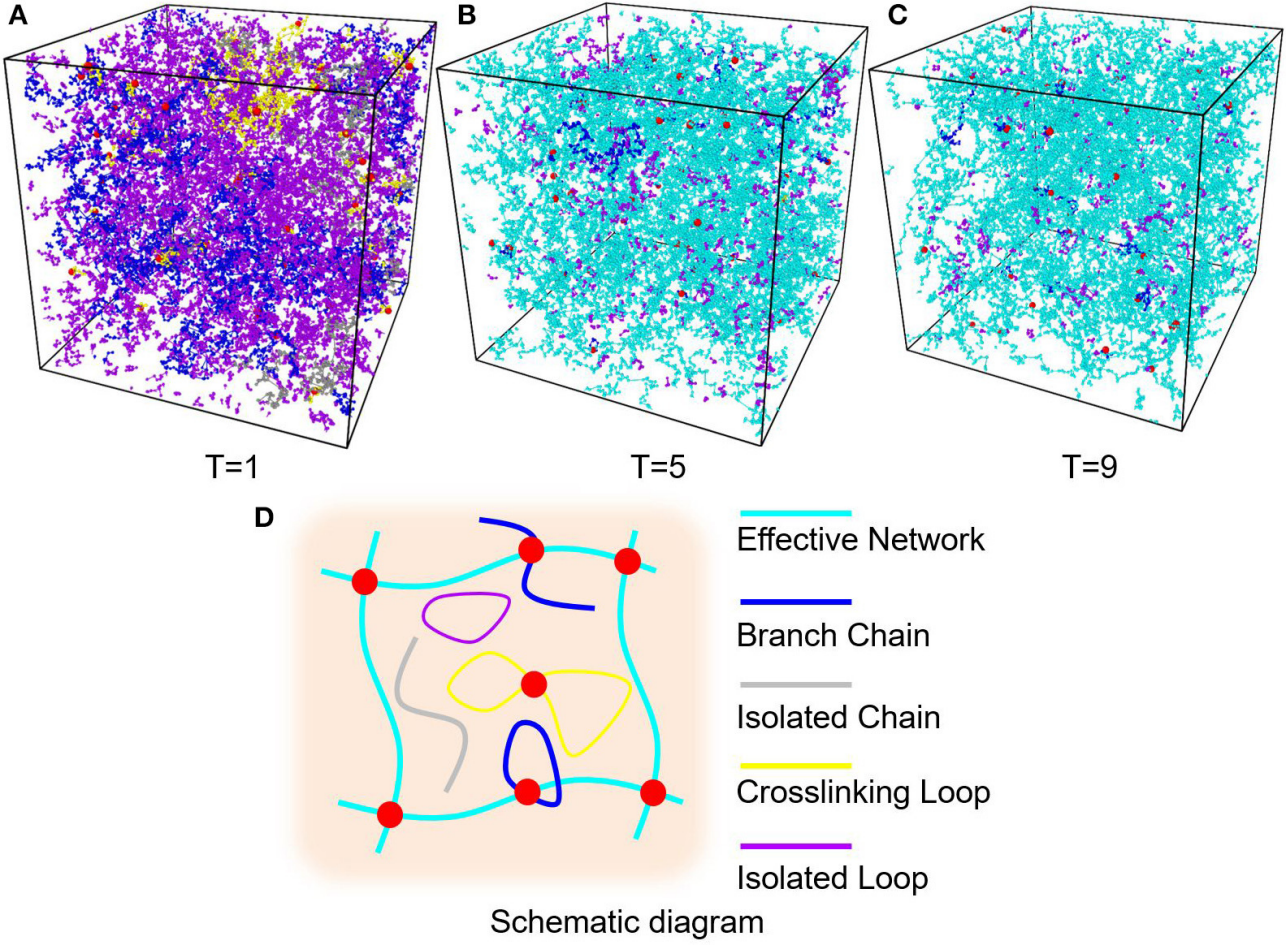
**(A)** PAAm hydrogel models crosslinking when *T* = 1. **(B)** PAAm hydrogel models crosslinking when *T* = 5. **(C)** PAAm hydrogel models crosslinking when *T* = 9. **(D)** Schematic diagram of the different types of chains in PAAm hydrogel (Cyan chains are effective network chains. Blue chains are branch chains. Gray chains are isolated chains. Yellow chains are crosslinking loops. Magenta chains are isolated loops).

In order to find a proper temperature for crosslinking simulations, six temperatures, i.e., *T* = 1, 3, 5, 7, 9, 11, is used to control the diffusion length of precursor beads in the crosslinking simulations. Figures 5A–C show part of the PAAm hydrogel models after crosslinking under temperatures *T* = 1, 5, 9, respectively. To analyze the generated complex structure of PAAm hydrogel models, polymer chains in all models are classified into five types, i.e., effective network chains, branch chains, isolated chains, crosslinking loops and isolated loops, as the schematic diagram shown in Figure 5D. Effective network chains form the crosslinking network throughout the hydrogel model. Branch chains attach to the effective network. It may be a chain with one free end or a loop. Isolated chains, crosslinking loops and isolated loops have no covalent bonds with effective network. The number and length of each type of chains are counted and averaged between the same groups of models. The crosslinking rate can be obtained by *N*_*bond*_/(*N*_*AAm*_ + 2*N*_*MBAA*_), where *N*_*bond*_ is the total bond number, *N*_*AAm*_ the number of AAm beads, *N*_*MBAA*_ the MBAA beads, and *N*_*AAm*_ + 2*N*_*MBAA*_ the maximum bonds between these monomers and crosslinkers. The final crosslinking rate for all models is above 99.97%. This indicates the crosslinking process in every model is sufficient. Chain numbers and number fractions for different type of chains are counted for all models. The chain lengths for different type of chains are counted as the bond number in current chain. The chain length fraction is calculated by the current chain length over total chain length.

**Figure 6:**
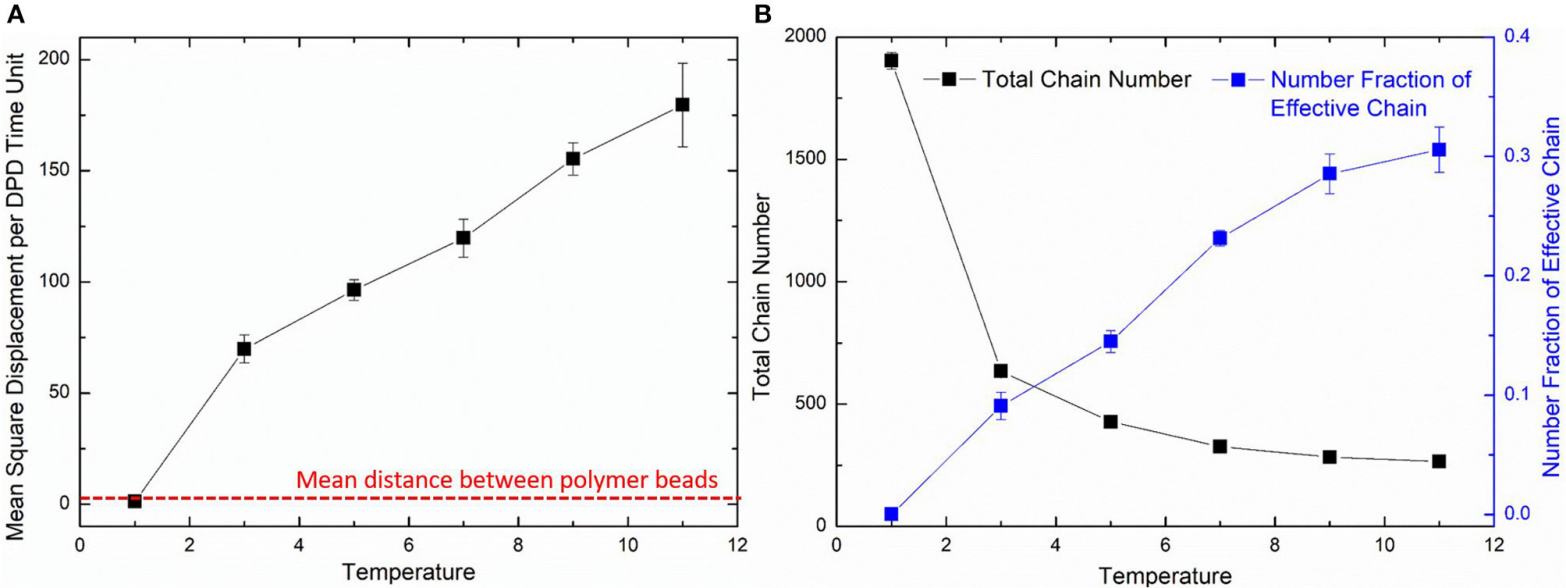
**(A)** The diffusion length of polymer beads in different temperatures. **(B)** The total chain number and the number fraction of effective chains in PAAm hydrogel models crosslinking under different temperatures.

**Figure 7:**
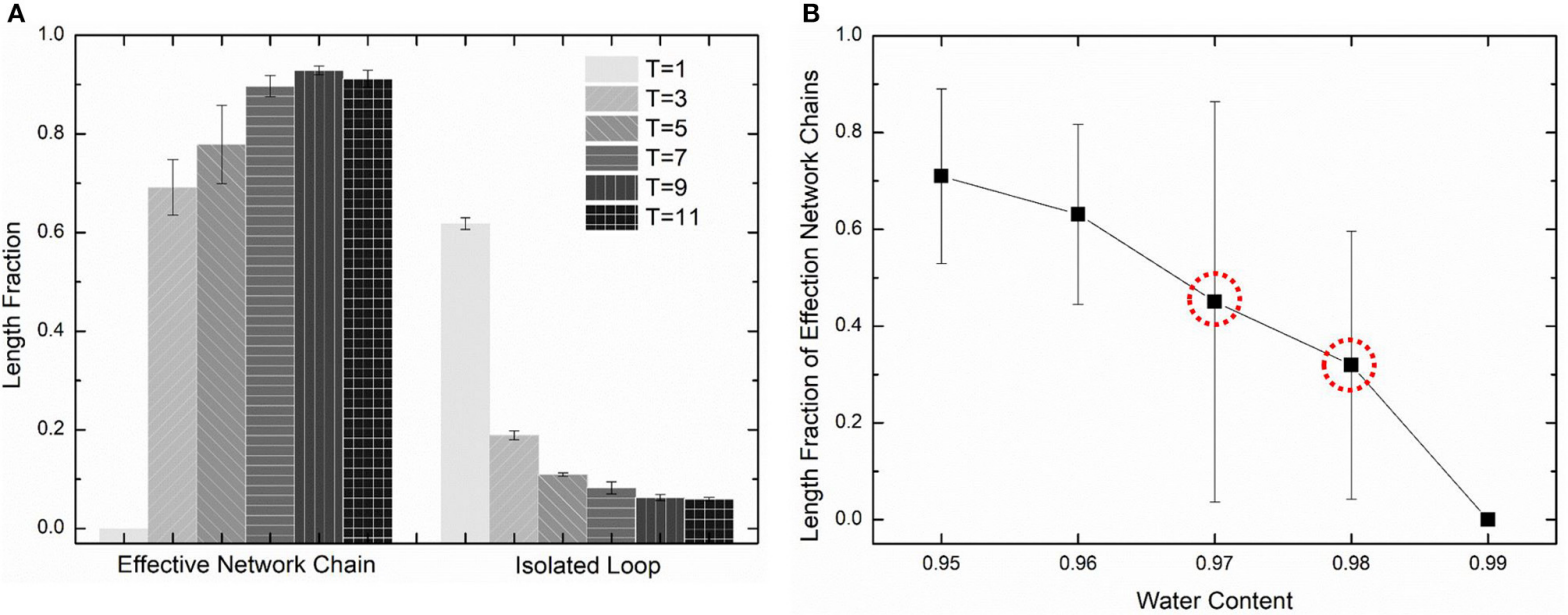
**(A)** The length fraction of effective network chains and isolated loops in PAAm hydrogel models crosslinking under different temperature. **(B)** The length fraction of effective network chains in PAAm hydrogel models crosslinking under different water contents.

The diffusion length of polymer beads is the key to the formation of polymer network. Figure 6A shows the diffusion length per DPD time unit in different temperatures. When *T* = 1, the diffusion length per DPD time unit is 1.13*R*_*c*_. This is even lower than the mean distance between polymer beads 1.52*R*_*c*_ marked as red dashed line in Figure 6A. It makes the precursor solution fail to form polymer network as shown in Figure 5A. Polymer beads crosslinking with neighbors form large amount of isolated loops with short length, corresponding to the largest chain number as shown in Figure 6B and the highest length fraction of isolated loops. With the increase of temperature from 3 to 11, the diffusion length per DPD time unit is much larger than the mean distance of polymer beads as shown in Figure 6A. Polymer network forms in crosslinking process. Both the number fraction and the length fraction of effective network chains shown in Figures 6B, 7A rise up with the temperature increasing. The length fraction of effective network chains reach to the top when *T* = 9. Meanwhile, the total chain number and the length fraction of effective chains and isolated loops tends to converge when temperature increases. This convinces us that our crosslinking simulations are sufficient in high temperatures and our PAAm hydrogel models are reliable. We also note that in Figure 7A the length fraction of isolated loops converges to about 7% with the temperature increasing. It indicates that the larger diffusion length, which may results from higher temperature, longer gelation time or more sufficient stirring in experiments, cannot eliminate the formation of isolated loops. The success of forming polymer network proves that our crosslinking simulation is a practical way to construct PAAm hydrogel model.

Further validation of our crosslinking method are conducted by predicting the crosslinking limit of the polymer content. Random mixture models with five water content 99, 98, 97, 96, and 95% are crosslinking with the temperature *T* = 9. Figure 7B shows the effective chain ratio of models with different water contents. It can be seen that models with water content 99% fail to form polymer network. Meanwhile, one of the three models with water content 97% also fails to form polymer network as well as one of the three models with water content 98%. These simulations give the crosslinking limit of water content 97%, which is very close to the swelling limit of many PAAm hydrogels in experiments (Zhang et al., 2018). The prediction of crosslinking limit also proves the validity of our crosslinking simulations and PAAm hydrogel models.

**Figure 8:**
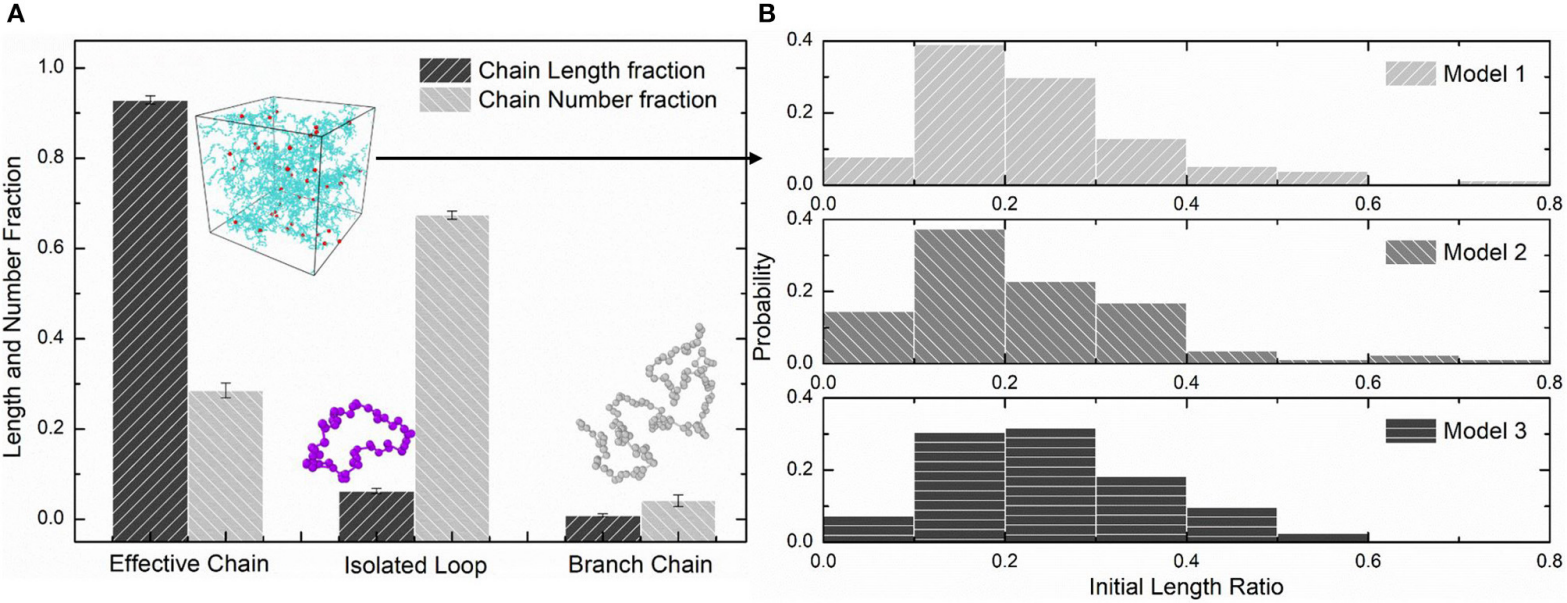
**(A)** The length fraction and number fraction of different types of chains in PAAm hydrogel models crosslinking under *T* = 9. **(B)** The distribution of the initial length ratio of the effective network chains in three PAAm hydrogel models crosslinking under *T* = 9.

**Figure 9:**
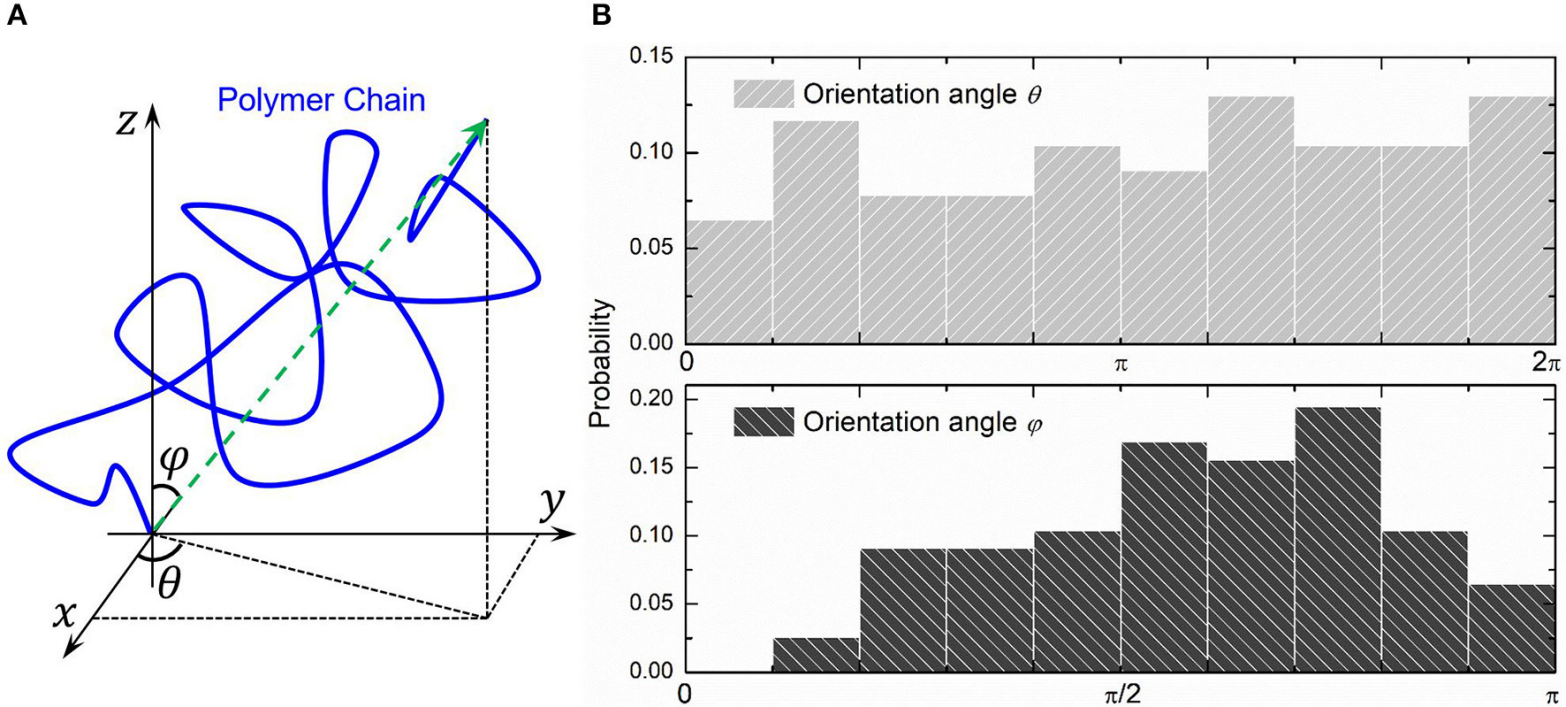
**(A)** The orientation angles (θ, φ) of a polymer chain. **(B)** The probability distribution of two orientation angles of all effective network chains.

We choose three PAAm hydrogel models with water content 80% crosslinking at *T* = 9 for further discussion. Figure 8A shows the number fraction and the length fraction of all types of chains in these models. Error bars for both the number fraction and length fraction indicate that our crosslinking process is reproducible. We denote all chains except effective network chains as ineffective chains. Only 7.1 ± 0.9% of polymer beads form ineffective chains, including branch chains 0.9 ± 0.4% and isolated loops 6.2 ± 0.6%. Because the mean contour length of ineffective chains 8.8 ± 0.7*r*_0_ is much lower than the effective network chains 288.7 ± 10.5*r*_0_, the length fraction of effective chains is 92.9 ± 0.9% though the number fraction is 28.5 ± 1.7%. Such high mean contour length leads to compliant effective network with the chain conformation more winding than other full-atom models (Tönsing and Oldiges, 2001; Oldiges and Tönsing, 2002; Wu et al., 2009; Mathesan et al., 2016; Xu et al., 2016; An et al., 2019; Hou et al., 2019) and coarse-grain models (Nawaz and Carbone, 2014; Wei et al., 2017; Xing et al., 2019) of PAAm hydrogels. This mean contour length is very close to that estimated by *N*_*AAm*_:2*N*_*MBAA*_=271 from precursor ratio. The mean end-to-end distance of effective network chains is 47.0 ± 3.6*r*_0_. The distributions of the initial length ratio which is the end-to-end distance over the contour length of each effective network chain in three models are shown in Figure 8B. It can be seen that the initial length ratio of effective chains shows nearly a Maxwell distribution. In order to validate the statistical properties of our models, we also present the distribution of two orientation angles (θ, φ in Figure 9A) and the ratio of the end-to-end distance to the contour length of all effective network chains in Figure 9B. The distribution of the chain orientation angle θ is almost uniform and the distribution of the chain orientation angle φ is almost sine-shaped. It proves that our model is sufficiently large to bridge the molecular and continuum properties of PAAm hydrogel.

Water beads are uniformly distributed around polymer chains. Water molecules in hydrogels are trapped by hydrophilic functional groups in polymer network via hydrogen bonds. The coverage of these hydrogen bonds is only within several water molecules away, meaning that the so-called bound water in hydrogels should be distributed uniformly surrounding polymer network and other chains. The hydrophilia of the polymer beads is embodied in our simulation as the relatively small Flory-Huggins parameter χ. It can be seen that our PAAm hydrogel models give reasonable crosslinking threshold and show sufficiently realistic and large polymer network in PAAm hydrogel. It convinces us to use these models to investigate the structural and mechanical properties of PAAm hydrogel.

### Large Deformation Behavior

In this section, Uniaxial tension tests with constant volume with $\lambda_{1}=\lambda ,\lambda_{2}=\lambda_{3}=\lambda^{-\frac{1}{2}}$, where λ_*i*_ is the stretch in i-th direction, are performed in DPD simulations to investigate the large deformation behavior of PAAm hydrogels with water content 80%. The nominal stress of the incompressible uniaxial tension test is obtained from

$$\begin{array}{l} P_{11}=\frac{{2\sigma }_{1}-\sigma_{2}-\sigma_{3}}{2\lambda }\; \end{array}$$

where σ_*i*_ is the Virial stress in each direction.

**Figure 10:**
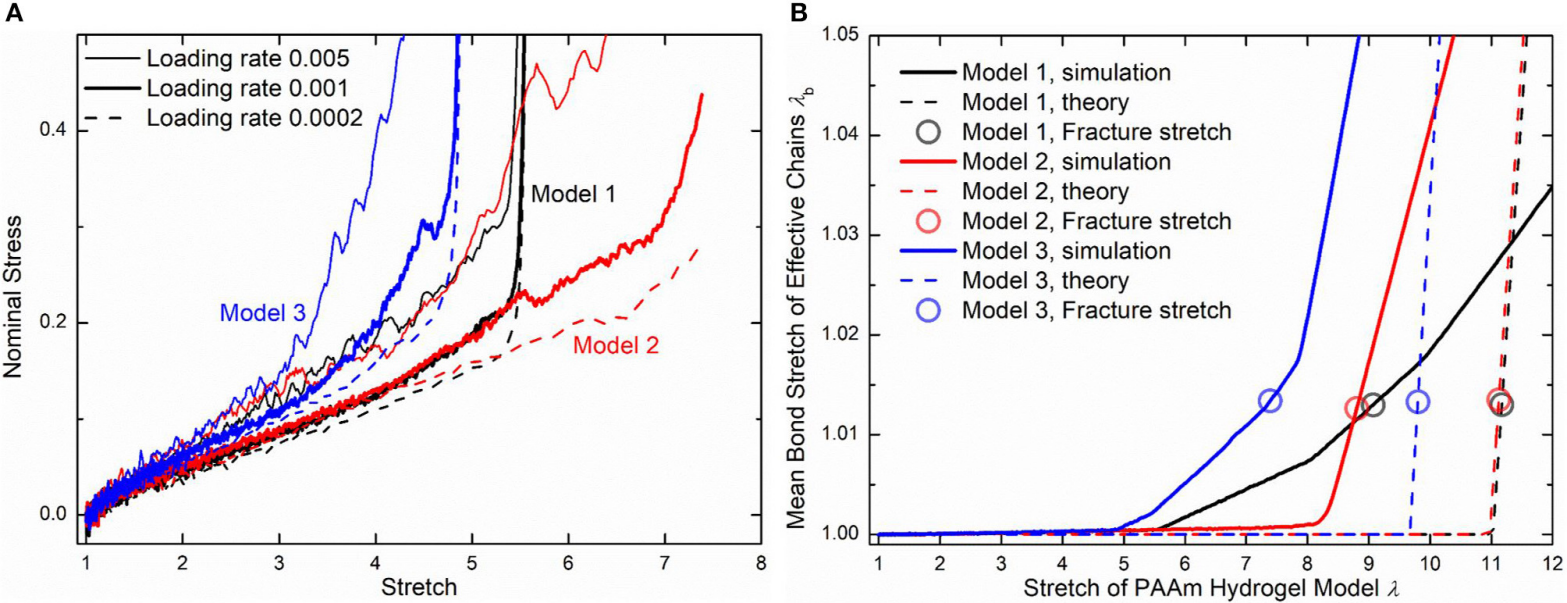
**(A)** The nominal stress-stretch curves of three PAAm hydrogel models under uniaxial tension tests with three loading rates 0.005, 0.001, and 0.0002. **(B)** Mean bond stretch vs. the stretch of PAAm hydrogel model curves (Solid lines are the mean bond stretch of PAAm hydrogel models vs. model stretch curves. Dashed lines are the theoretical mean bond stretch vs. model stretch curves. Dots are the fracture point. Model 1, 2, and 3 are three independent PAAm hydrogel models with the water content 80%. Data for Mode 1, 2, and 3 are colored as black, red, and blue).

Three loading rates, i.e., 0.005, 0.001, and 0.0002 per DPD time unit, are imposed on PAAm hydrogel models. Figure 10A shows the nominal stress-stretch curves of three PAAm hydrogel models under three loading rates. It shows that our PAAm hydrogel models capture both the viscoelastic and the hyperelastic behaviors. All three models shows the loading rate-dependent behavior. This is because the viscosity of all beads is embedded in DPD force field *F*^*D*^. Higher loading rate causes more intensive rebound of bonded beads, leading to the higher dissipative force. Compared to previous experimental results (Lei et al., 2019; Yang et al., 2019), the loading rate-dependent behavior is much more significant since the loading rates we adopt in our DPD simulations are far larger than realistic ones used in experiments. Considering the loading rate-dependent behavior and the computational resources, we choose loading rate 0.001 per DPD time unit for further discussion. The hyperelasticity is shown in the initial part of the stress-stretch curve during uniaxial tension. The hardening stage can also be found where the stretch is large, which is cause by the stretch limit of polymer chains. We also present the bond stretch in effective chains during the uniaxial tension tests as the solid lines shown in Figure 10B. It shows that significant bond stretch occurs in the hardening stage where effective chains are stretched to almost straight. The trend of the nominal stress-stretch curves agrees well with experimental results (Bai et al., 2017; Zhang et al., 2018; Yang et al., 2019). The scattered stretch limit where hardening stage occurs in PAAm hydrogels with the same water content are also found.

Although the number fraction and length fraction of each type of chains in three PAAm hydrogel models only have a <2% standard deviation, their mechanical behaviors in Figure 10A show much differences, especially when the stretch is large. This is not what the current theory of hydrogels can predict. Therefore, we compare our simulation results with current constitutive theory of polymers to reveal the underlying mechanism of the large deformation behavior of PAAm hydrogel. In current constitutive theory (Huang et al., 2020), two key parts, i.e., the free energy of a single chain and the statistical sum of the free energy of all chain, are crucial to bridge the mesoscale chain conformation change to the bulk mechanical response of hydrogels. Considering the polymer chains in our DPD simulations are close to the so-call freely joint chains, the Langevin chain model is adopted to characterize the free energy of a single chain

$$\begin{array}{l} W_{chain}=kTN\left(\lambda_{c}l_{0}\beta +\ln\frac{\beta }{\sinh\beta }\right),\;\beta =L^{-1}\left(\lambda_{c}l_{0}\right)\; \end{array}$$

where *kT* is the thermodynamics energy unit, *N* the number of Kuhn segments in current chain, λ_*c*_ the stretch of chain with respect to the mean initial length ratio of current chain $l_{0}=\frac{L_{0}}{N_{b}b_{0}}$, *L*_0_ the mean end-to-end distance of current chain, *N*_*b*_*r*_0_ the mean contour length with the mean bond number *N*_*b*_ and the bond length *r*_0_. Since the inverse Langevin function $L^{-1}$ is singular when λ_*c*_*l*_0_ = 1, the Langevin model often need additional modification (Mao et al., 2017). Li and Bouklas (2020) proposed the stretching force of a polymer chain caused by the conformation entropy change can also stretch bonds in polymer chains. Thus, the modified chain stretch λ_*c*_ turns out to be λ_*c*_/λ_*b*_, where λ_*b*_ is the mean stretch of bonds in effective chains. Combining with the bond force *F*^*B*^ in section Governing Equation of DPD Simulations, this modification circumvents the singularity of the inverse Langevin function by giving the relationship of chain stretch λ_*c*_ to the mean bond stretch λ_*b*_ as follows

$$\begin{array}{l} \lambda_{c}=\frac{\lambda_{b}}{l_{0}}L\left(\frac{N_{b}^{2}}{N}\frac{C}{kT}\lambda_{b}\left(\lambda_{b}-1\right)\right)\; \end{array}$$

In order to statistically sum up the free energy of all effective chains, the mean stretch of all effective chains $\langle \lambda_{c}\rangle =\sqrt{I_{1}/3}$ and mean number of Kuhn segments 〈*N*〉 are used to formulate the total free energy of hydrogel model as follows

$$\begin{array}{l} W=nkT\langle N\rangle \left(\sqrt{\frac{I_{1}}{3}}\frac{l_{0}}{\lambda_{b}}\beta +\ln\frac{\beta }{\sinh\beta }\right),\;\beta =L^{-1}\left(\sqrt{\frac{I_{1}}{3}}\frac{l_{0}}{\lambda_{b}}\right)\; \end{array}$$

where *n* is the effective chain density. *I*_1_ is the first invariant of the deformation gradient of bulk hydrogel model with $I_{1}=\lambda_{1}^{2}+\lambda_{2}^{2}+\lambda_{3}^{2}=\lambda^{2}+{2\lambda }^{-1}$, which connect the chain conformation change in mesoscale to the bulk deformation in continuum. Thus, the λ_*b*_ − λ relationship between mean bond stretch and the stretch of PAAm hydrogel models can be obtained theoretically by solving following equation

$$\begin{array}{l} \sqrt{\frac{I_{1}}{3}}=\frac{\lambda_{b}}{l_{0}}L\left(\frac{N_{b}^{2}}{N}\frac{C}{kT}\lambda_{b}\left(\lambda_{b}-1\right)\right)\; \end{array}$$

**Figure 11:**
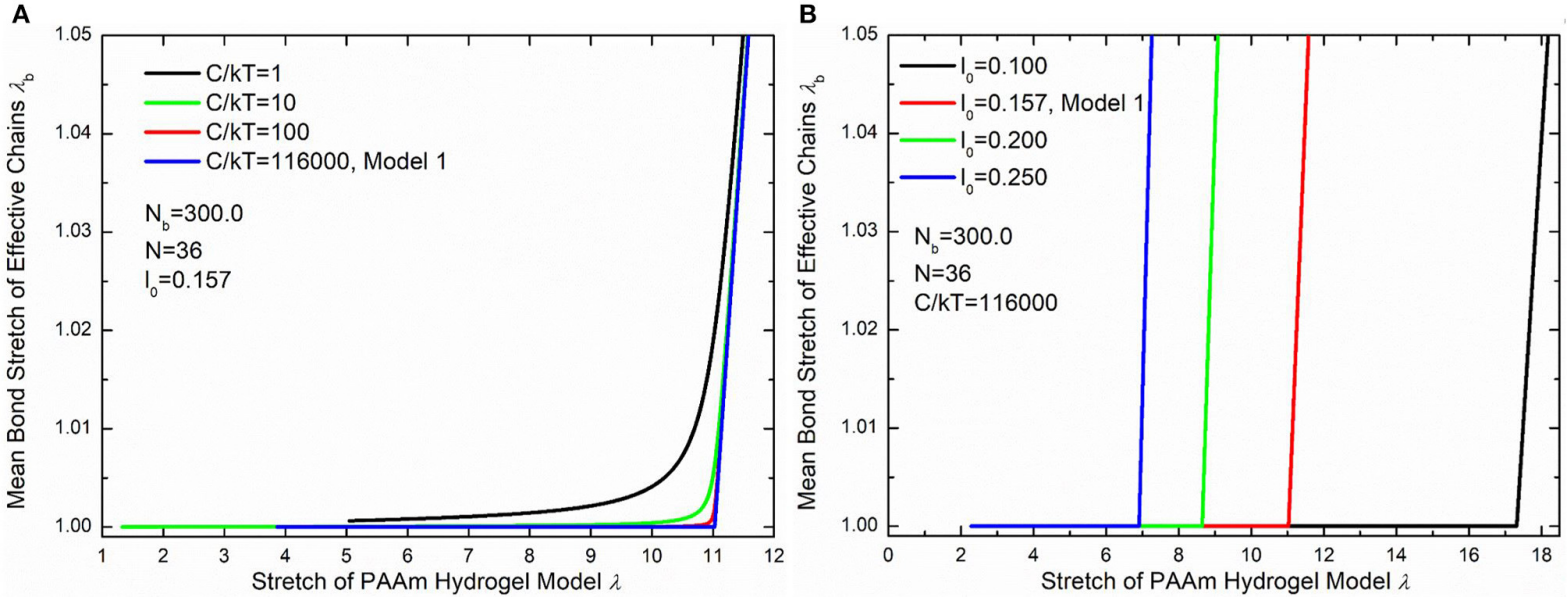
Parameter study of the λ_*b*_ − λ relationship. **(A)** The λ_*b*_ − λ relationship with different bond strengths. **(B)** The λ_*b*_ − λ relationship with different initial length ratios.

**Figure 12:**
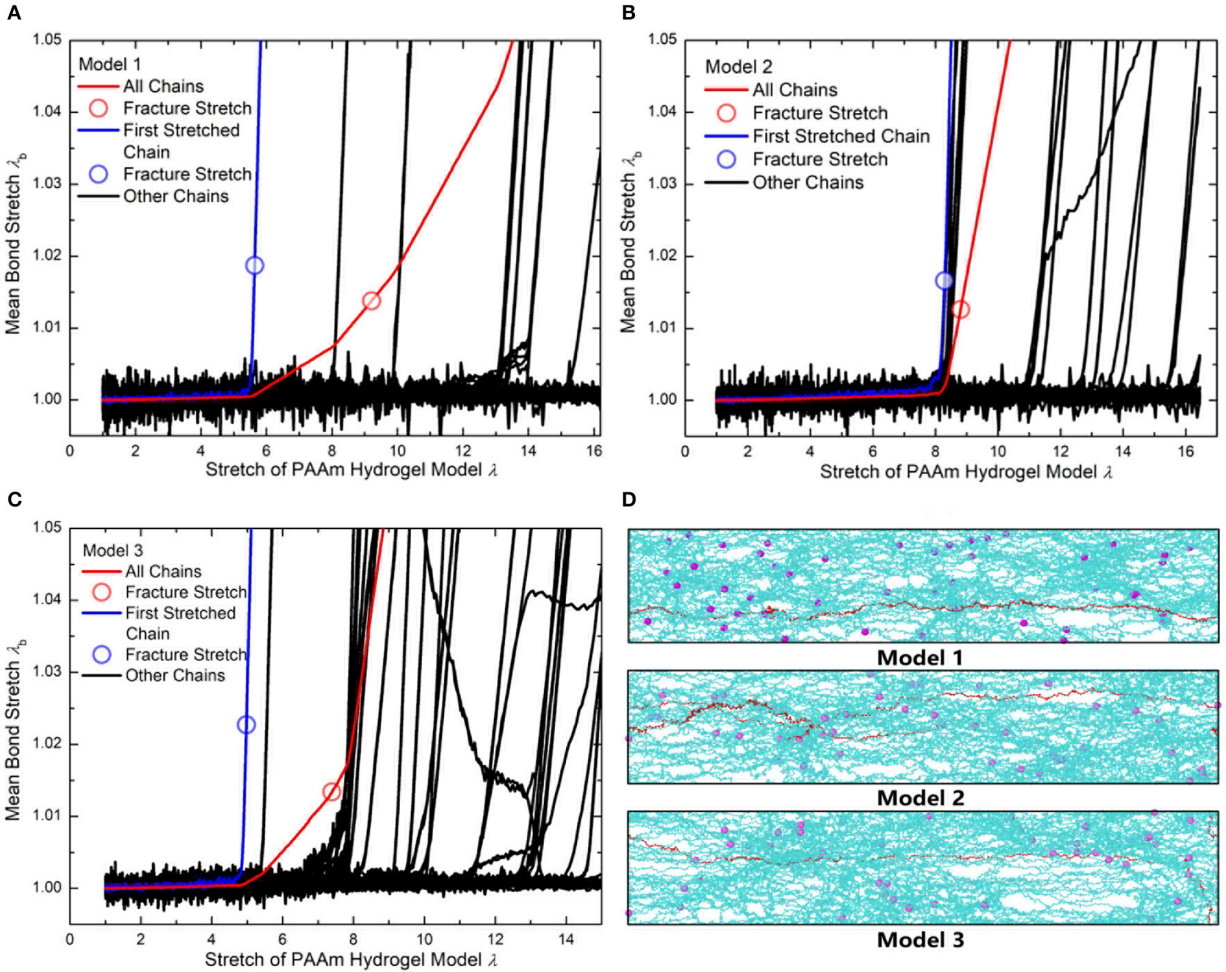
**(A)** The mean bond stretch of every effective chains and the total mean bond stretch vs. stretch of Model 1. **(B)** The mean bond stretch of every effective chains and the total mean bond stretch vs. stretch of Model 2. **(C)** The mean bond stretch of every effective chains and the total mean bond stretch vs. stretch of Model 1 (Blue line is the first polymer chain stretched to fracture in each model. Red line is the total mean bond stretch vs. model stretch in each model. Black lines are the mean bond stretch of the rest effective chains in each model). **(D)** The configuration of three models when model stretch is 3.7 (Cyan lines are effective chains. Purple particles are MBAA beads. Red line is the first effective chain stretched to fracture in each model).

Figure 10B compare the theoretically λ_*b*_ − λ relationship with that measured in simulations. It is very clear that there are large discrepancies between theoretical predictions and simulation results. Parameter study for the λ_*b*_ − λ relation is performed to find the reason for the large discrepancies. Figures 11A,B shows the λ_*b*_ − λ relationship with the different bond strength parameter $\frac{C}{kT}$ and different mean initial length ratio *l*_0_, respectively. Combining Figures 10B, 11A, we can find that the simulation results show a much softer mean bond stretching process during uniaxial tension of PAAm hydrogel models than theoretical predictions, although such a large bond strength parameter $\frac{C}{kT}=$ 116,000 is used in both simulations and theory. From lines in Figures 12A–C, we can find that the softer mean bond stretch of the whole model is moderated by the unsynchronized stretch of different effective chains. Also, the flat stress-stretch curves for hydrogel actually result from the superposition of the unsynchronized stiff bond stretching behavior of large amount of chains. In Figure 11B, we can find that the critical stretch where significant bond stretch occurs depends on the initial length ratio. The discrepancy of critical stretch between theory and simulation is because of the use of mean initial length ratio. As shown in Figure 8B, the initial length ratio of effective chains distributes in a very wide range. Chains with high initial length ratio first stretch to almost straight, so that using the lower mean initial length ratio overestimates the critical stretch in Figure 11B.

The nominal stress-stretch curves and the λ_*b*_ − λ relation indicates that current continuum constitutive theory for hydrogel is only valid in small stretch, while the mechanical response under large deformation near fracture needs detailed structural models. The statistical averaging structural properties, such as water content and mean chain length, smears out the wide distribution of the possible conformation of effective chains, while specific structural features are what dominate the large deformation behavior of hydrogel.

### Fracture Criterion

The randomness of the polymer network in hydrogel makes it hard to give an accurate fracture criterion. The widely used fracture toughness works poor as the fracture criterion of hydrogels since it often counts the deformation energy far away from the crack. As shown in the PAAm hydrogel models, the stretch limit is the key property that determines the fracture of a polymer chain. A stretch criterion is more general which can be applied in different polymer network systems and more practical for different loading conditions. In this section, we propose a stretch criterion of the fracture of hydrogels.

The critical energy to the fracture of a polymer chains is equal to the fracture energy of one C-C bond

$$\begin{array}{l} W_{cr}=\frac{C}{2}r_{0}^{2}{\left(\lambda_{b}^{cr}-1\right)}^{2}\; \end{array}$$

where $\lambda_{b}^{cr}$ is the critical stretch when a C-C bond breaks. This critical free energy corresponds to the critical mean stretch of all bonds ${\bar{\lambda }}_{b}^{cr}$ in this chain with

$$\begin{array}{l} W_{cr}=\frac{N_{b}C}{2}r_{0}^{2}{\left({\bar{\lambda }}_{b}^{cr}-1\right)}^{2}\; \end{array}$$

Thus, the fracture criterion for a single chain can be expressed as

$$\begin{array}{l} {\bar{\lambda }}_{b}^{cr}=1+\frac{\lambda_{b}^{cr}-1}{\sqrt{N_{b}}}\; \end{array}$$

Using the ${\bar{\lambda }}_{b}^{cr}=$1.225 when *N*_*b*_ = 2 obtained in Figure 4, the critical stretch of a C-C bond $\lambda_{b}^{cr}$ is 1.318. The critical mean stretch of a C-C bond $\lambda_{b}^{cr}$ is then related to the first invariant of the bulk deformation gradient by substituting the critical mean stretch ${\bar{\lambda }}_{b}^{cr}$ into the λ_*b*_ − λ relation. As the dots shown in Figure 10B, for three models with the *N*_*b*_ = 600.0, 558.6, 573.6, the theoretical fracture stretch are 11.2, 11.1 and 9.8, and the fracture stretch measured from simulations are 9.1, 8.8, and 7.4, respectively. There are much discrepancies between the theoretical results and simulation results, mainly because the equal-chain-length assumption in current constitutive theory overestimates the mean chain stretch by $\langle \lambda_{c}\rangle =\sqrt{I_{1}/3}$. Furthermore, the fracture stretch measured from simulations using the mean bond stretch of all effective chains should be the upper limit of the real fracture stretch. Considering the unsynchronized stretch of different effective chains, 50% of effective chains may have been broken when the mean bond stretch of all effective chains reaches fracture stretch. This amount of chain scission is enough for the macroscopic fracture of hydrogel. On the other hand, we can get the lower limit of the fracture stretch by analyzing the fracture stretch of every effective chains. Blue dots in Figures 12A–C are the fracture stretch where the first effective chain (as shown in Figure 12D) is about to break, while red dots are fracture stretch obtained from mean bond stretch of all effective chains. It gives limited ranges of the fracture stretch of our three PAAm hydrogel models as 5.6–9.1, 8.3–8.8, and 5.0–7.4, respectively. These ranges almost cover the fracture stretch of PAAm hydrogels with the water content 78% in previous experiments (Zhang et al., 2018) as 5.5, 8.6 and 6.1.

Our coarse-grain PAAm hydrogel models are the representative models for numerous hydrogels since only the repulsive coefficient *a* is adjustable for different hydrogel system. Hydrogels share the same large deformation mechanism with the conformation change of the complex polymer network, so that the detailed mesoscale model is such a powerful tool to bridge the molecular movement to bulk deformation.

## Conclusions

In this study, we propose a method to construct the mesoscale PAAm hydrogel models and investigate the large deformation and fracture mechanism of PAAm hydrogel using DPD simulations. The coarse-grain PAAm hydrogel models are constructed by simulating the crosslinking process in experiments. Different temperatures are tested for achieving sufficient diffusion length of polymer beads for sufficient crosslinking. It shows that the formation of polymer network in crosslinking process only occurs when the diffusion length of polymer beads is much larger than the mean distance of polymer beads. However, the increasing of diffusion length, which may caused by the increasing of temperature, gelation time or stirring in experiments, cannot eliminate the formation of isolated loops. Our PAAm hydrogel models have realistic structure of polymer network, including the compliant effective network, branch chains, isolated chains, crosslinking loops and isolated loops. The degree of polymerization of the effective network chains is almost the same to the theoretical estimation. Our crosslinking simulations also show the upper limit of the water content for forming polymer network is about 97% which is close to the swelling limit of PAAm hydrogel in experiments. The scale of our PAAm hydrogel models are proved to be sufficiently large by the uniformly distributed orientation of effective chains. Incompressible uniaxial tension tests are performed in three different loading rates using our PAAm hydrogel models with the water content 80%. The nominal stress-stretch curves reflect both the hyperelasticity and the viscoelasticity of our PAAm hydrogel models. However, the scattered large deformation behaviors of PAAm hydrogel models with the same water content indicate that the mesoscale conformation of polymer network have great impact on the mechanical behavior in large stretch, because the effective chains with a wide range of initial length ratio stretch to straight at different time during deformation. Furthermore, we propose a stretch criterion of the fracture of PAAm hydrogel using the fracture stretch of C-C bonds. By analyzing our PAAm hydrogel models, specific upper and lower limit of the fracture stretch are given for each PAAm hydrogel models. These ranges agree quite well with experimental results. Our coarse-grain PAAm hydrogel models can be applied to numerous single network hydrogel systems.

## Data Availability Statement

All datasets generated for this study are included in the article/Supplementary Material.

## Author Contributions

JL, SX, and ZLi: investigation. ZLiu: resources, supervision, and funding acquisition. JL and SX: writing—original draft preparation. ZLi and ZLiu: writing—review and editing.

### Conflict of Interest

The authors declare that the research was conducted in the absence of any commercial or financial relationships that could be construed as a potential conflict of interest.
