# Supplementary material for: Study on Large Deformation Behavior of Polyacrylamide Hydrogel Using Dissipative Particle Dynamics
Source: Front Chem. 2020 Feb 25;8:115. doi: 10.3389/fchem.2020.00115 (PMC7052281; doi:10.3389/fchem.2020.00115)
Supplement: Supplementary file 1 [file Data_Sheet_1.PDF]

Supplementary Material

1 Supplementary Data

Consistent valence force field (CVFF) is used to simulation the full-atom AAm chain model in Section 2.4. The atomic interaction is composed of Lennard-Jones potential, electrostatic interaction, morse bond potential, harmonic bond angle potential, bond dihedral potential and harmonic bond improper potential as follows

$$E = 4\varepsilon\left[\left(\frac{\sigma}{r}\right)^{12} - \left(\frac{\sigma}{r}\right)^{6}\right] + \frac{Cq_iq_j}{\epsilon r} + D\left[1 - e^{-\alpha(r-r_0)}\right]^2 + K_1(\theta-\theta_0)^2 + K_2[1+\cos(n\phi - d)] + K_3(\chi-\chi_0)^2$$

with the coefficients shown in Table S1-S5.

Table S1 Non-bond coefficient

Atom type	Relative Mass	Charge	ε(eV)	σ(Å)
C(sp^3)	12.0107	-0.2	0.16	3.4745
C(sp^3)	12.0107	-0.1	0.16	3.4745
C(sp^2)	12.0107	0.38	0.148	3.6170
O	15.9996	-0.38	0.228	2.8598
N	14.006	-0.56	0.167	3.5012
H	1.008	0.1	0.038	2.45
H (-NH_2)	1.008	0.28	0.038	2.45

Table S2 Bond coefficient

Bond type	D(eV)	α	r_0(Å)
C-C	88	1.915	1.526
C-H	108.6	1.771	1.105
C=O	145	2.06	1.23
C-N	97	2	1.32
N-H	93	2.28	1.026

Table S3 Bond angle coefficient

Angle type	K_1(eV)	θ_0(DEG)
H-C-H	39.5	106.4
C(sp^3)-C(sp^3)-X	44.4	110
C(sp^2)-C(sp^3)-H	46.6	110.5
O=C-N,O=C-C	68	120
C(sp^2)-N-H	53.5	114.1
H-N-H	37.5	115

Table S4 Bond dihedral coefficient

Dihedral type	K_2(eV)	n	d	Weight factor
X-C(sp^3)-C(sp^3)-X	1.4225	3	0	0
X-C(sp^3)-C(sp^2)-X	0	0	0	0
X-C(sp^2)-N-X	6	2	180	0

Table S5 Bond improper coefficient

Improper type	K_3(eV)	χ_0(DEG)
C-C=O -N	24.3329	0
